# A Novel Analytical Modeling Approach for Quality Propagation of Transient Analysis of Serial Production Systems

**DOI:** 10.3390/s22062409

**Published:** 2022-03-21

**Authors:** Shihong Liu, Shichang Du, Lifeng Xi, Yiping Shao, Delin Huang

**Affiliations:** 1 Department of Industrial Engineering and Management, School of Mechanical Engineering, Shanghai Jiao Tong University, Shanghai 200240, China; liukang1644@sjtu.edu.cn (S.L.); lfxi@sjtu.edu.cn (L.X.); 2 College of Mechanical Engineering, Zhejiang University of Technology, Hangzhou 310023, China; syp123gh@zjut.edu.cn; 3 College of Mechanical Engineering, Donghua University, Shanghai 201620, China; huangdelin@dhu.edu.cn

**Keywords:** production systems, transient analysis, quality, bottleneck, Markov models

## Abstract

Production system modeling (PSM) for quality propagation involves mapping the principles between components and systems. While most existing studies focus on the steady-state analysis, the transient quality analysis remains largely unexplored. It is of significance to fully understand quality propagation, especially during transients, to shorten product changeover time, decrease quality loss, and improve quality. In this paper, a novel analytical PSM approach is established based on the Markov model, to explore product quality propagation for transient analysis of serial multi-stage production systems. The cascade property for quality propagation among correlated sequential stages was investigated, taking into account both the status of the current stage and the quality of the outputs from upstream stages. Closed-form formulae to evaluate transient quality performances of multi-stage systems were formulated, including the dynamics of system quality, settling time, and quality loss. An iterative procedure utilizing the aggregation technique is presented to approximate transient quality performance with computational efficiency and high accuracy. Moreover, system theoretic properties of quality measures were analyzed and the quality bottleneck identification method was investigated. In the case study, the modeling error was 0.36% and the calculation could clearly track system dynamics; quality bottleneck was identified to decrease the quality loss and facilitate continuous improvement. The experimental results illustrate the applicability of the proposed PSM approach.

## 1. Introduction

Production system modeling (PSM) is the process of mapping system principles between fundamental component-level elements (e.g., machine reliability, quality failure, and repair probability) and their impacts on system-level performance measures (e.g., quality and throughput). PSM is critical for analysis, disclosure, and understanding of production procedure principles for quality improvement. For example, General Motors implemented PSM at more than 30 plants, such as system performance estimation, bottleneck identification, and resource allocation optimization. As a result, General Motors improved revenue and saved more than USD 2.1 billion.

The literature on PSM, regarding quality propagation, mainly consists of two research lines. Traditionally, the research line focuses on the fundamental physical law. For instance, the state space models are established in a pioneering paper by Jin and Shi [1], linking the engineering knowledge for sources of variations with final product quality measures. More extensions of state space models are introduced to the three-dimensional assembly system [2] and machining system [3,4,5]. Although state space models are still popular, essential problems exist for this research. Namely, the state space models rely on complex production process kinematics and only apply to dimension errors that impede further applicability.

In another research line, there arises a prevailing trend of mapping the correlation between production systems and quality propagation based on Markov analytical models. Related studies indicate that the production system has a strong impact on system quality performance. Zhao et al. [6] introduced a Markov model of flexible production lines with setups using recursive procedures. Du, Xu, and Li [7] expanded a discrete Markov model to multi-type product systems with calculations of transition probabilities to obtain quality probability. Şahin et al. [8] applied the Markov model to capture the incoming batch quality and they predicted the future arrival quality. Goswami, Kumar, and Ghadge [9] determined steady-state probabilities using the Markov model and discussed the guidance. Guo and Gu [10] formulated a mixed Markov model of production and maintenance to evaluate the system quality under optimal policies. Yaghoubi et al. [11] introduced quantitative tools using the Markov method and improved the quality of the oil product line. The Markov model applications also include battery production systems [12], machine tool modeling [13], k-out-of-n systems [14,15], reconfigurable production systems [16], maintenance policy [17], re-entrant production systems [18], primary care [19], rolling bearing monitoring [20], industrial network models [21], assembly assistance systems [22], laser-based manufacturing process [23], and the steel production process [24,25].

In spite of the above efforts, it should be noted that most existing studies of PSM for quality are focused on steady-state analyses, which characterize long-term system quality behavior. Unlike these results, where a large amount of papers have been devoted to steady-state analysis, transient analysis for system quality receives much less attention and still needs further development. After the product changeover or maintenance activities, transients of system quality are typically encountered because of undesired initial system circumstances, such as relocating errors of flexible fixtures when a fresh process starts up. The system quality operates either partially or even entirely in transient regimes, which depicts the behavior of system quality before reaching steady-state at the desired criteria for quality and cost. The specific characteristics of the transient phase differ from steady-state phase significantly, resulting in associated quality loss. The management techniques appropriate for stable production cannot perform well in unstable transients. Similar scenarios also exist for many other production systems, for example aerospace, automobiles, vehicles, appliance, and electronics systems [26]. It is of significance to fully understand quality propagation during transients to shorten product changeover time, decrease quality loss, as well as improve quality. This paper intends to contribute to this end.

In the framework of transient analysis, some preliminary results have been reported in PSM of transient analysis for throughput. The main results of a transient throughput analysis can be categorized into two groups: computer simulation and analytical methods. Representative results of simulation can be found in [27,28]. The computer simulation approach is accurate, to capture system throughput during transients. However, high development costs, low flexibility, and long execution times of simulation models limit its capabilities. Representative results of analytical methods can be found in the paper by Wang, Huang, and Li [29], which investigated transient throughput properties of flexible systems with the finite buffer and Bernoulli machine. Further research includes a geometric serial line [30], assembly systems [31], a serial Bernoulli production line [32,33,34], and Bernoulli production systems with rework processes [35,36]. In contrast to computer simulation, analytical methods can inherently overcome the above drawbacks and disclose fundamentally mathematical coupling between performance measures and system parameters.

To summarize, there is increasing concern about PSM for quality performance and transient analysis of production systems. However, it is still needed to derive analytical methods, which integrate the two issues together. With such motivation, a novel analytical PSM approach was established to investigate product quality propagation for transient analysis of serial multi-stage production systems having remote quality information feedback (RQIF). Specifically, this approach overcomes the limited assumptions and restrictions of conventional quality models. Both quality corrections and quality degradations are addressed, which is more practical and accurate in a real production environment. Transient quality analyses were conducted to reveal the correlation between components and system quality performance. System theoretic properties of quality measures were analyzed and the quality bottleneck identification method was investigated.

The remainder of this paper is as follows. Problem assumptions are addressed and the Markov model is formulated to investigate quality propagation during transients in Section 2. In Section 3, the transient evaluation for quality performance of the two-stage systems is derived. Section 4 investigates multi-stage production systems and introduces an iterative procedure to approximate the transient quality performance. In Section 5, system theoretic properties of quality measures are analyzed. In Section 6, a case study is presented to verify the proposed PSM approach. Section 7 formulates the conclusions.

## 2. Problem Formulation and Modeling

### 2.1. Descriptive Models

**Figure 1:**
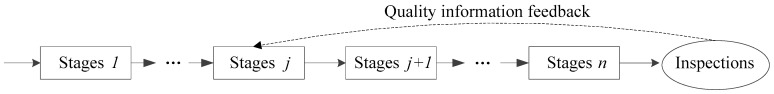
Multi-stage production systems having RQIF.

Assumptions for quality characteristics, inspection and system states transition of multi-stage production systems having RQIF are depicted (Figure 1).

**Figure 2:**
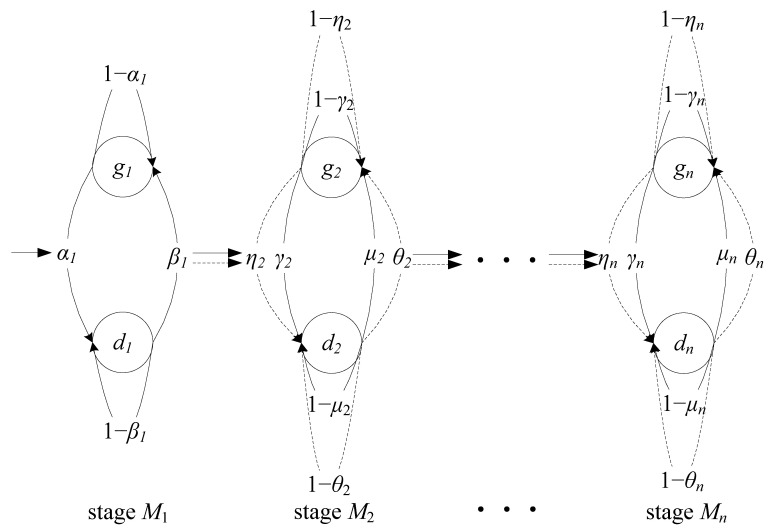
Diagrams of state transitions in multi-stage production systems.

1. The multistage production system is composed of n stages with the inspection station in the final stage.
2. The slots of the time axis are equal to the machine cycle time. Consider the working times of the production systems while the machine breakdown is not under consideration.
3. The product quality processed in stage $M_{i}\;\left(i\geq 2\right)$ depends on the quality state of stage $M_{i}$ and the incoming product quality from upstream stage $M_{i-1}$. Both quality corrections and quality degradations exist in production systems. The product could have better or worse quality after it is processed in a certain stage.
4. With respect to the quality state of stage $M_{i}$, denote stage $M_{i}$ as in the defective state $d_{i}$ or in the good state $g_{i}$ when stage $M_{i}$ produces a defective or good product in the time slot t.
5. With respect to the incoming product quality for stage $M_{i}$, it relies on the upstream stage $M_{i-1}$. Stage $M_{i-1}$ in the defective state $d_{i-1}$ or in the good state $g_{i-1}$ produces a defective or good product in the time slot $\left(t-1\right)$, indicating a defective or a good incoming product for stage $M_{i}$ in the time slot t, respectively.
6. When in the defective state $d_{1}$, stage $M_{1}$ may transition into a good state $g_{1}$ with a probability $\beta_{1}$ or transition into a defective state $d_{1}$ with $\left(1-\beta_{1}\right)$. When in a good state $g_{1}$, stage $M_{1}$ may transition into a defective state $d_{1}$ with probability $\alpha_{1}$ or transition into a good state $g_{1}$ with $\left(1-\alpha_{1}\right)$ (see Figure 2).

In case of a good incoming product, when in a defective state $d_{i}$, stage $M_{i}\;\left(i\geq 2\right)$ may transition into a good state $g_{i}$ with probability $\mu_{i}$ or transition into a defective state $d_{i}$ with $\left(1-\mu_{i}\right)$; when in a good state $g_{i}$, stage $M_{i}$ may transition into a defective state $d_{i}$ with probability $\gamma_{i}$ or transition into a good state $g_{i}$ with $\left(1-\gamma_{i}\right)$ (Figure 2).

In case of a defective incoming product, when in a defective state $d_{i}$, stage $M_{i}\;\left(i\geq 2\right)$ may transition into a good state $g_{i}$ with probability $\theta_{i}$ or transition into a defective state $d_{i}$ with $\left(1-\theta_{i}\right)$; when in a good state $g_{i}$, stage $M_{i}$ may transition into a defective state $d_{i}$ with probability $\eta_{i}$ or transition into a good state $g_{i}$ with $\left(1-\eta_{i}\right)$ (Figure 2).

Figure 2 depicts the diagrams of the state transition in multi-stage production systems. The solid lines with arrow between stages reflect good incoming products while the dashed lines reflect defective incoming products. Note that probabilities $\alpha_{1},\gamma_{i},\eta_{i}$ are denoted as quality failure probabilities, and $\beta_{1},\;\mu_{i},\;\theta_{i}$ quality repair probabilities. Systems with RQIF reflect the situations in which most, rather than all, operations are reliable for quality, while defective products are identified in the final stage. Such system examples are seen in semiconductor production, assembly systems, engines, and aircraft horizontal stabilizer assemblies.

### 2.2. Mathematical Models

Under assumptions 1–6, system transients and transient quality performance measures are derived for the two-stage systems and extended to multi-stage systems. The two-stage production system is described with four quality states as follows. (1) State $g_{1}g_{2}$ indicates that both $M_{1}$ and $M_{2}$ produce good parts; (2) state $g_{1}d_{2}$ indicates that $M_{1}$ produces good parts while $M_{2}$ produces defective parts; (3) state $d_{1}g_{2}$ indicates that $M_{1}$ produces defective parts while $M_{2}$ produces good parts; (4) state $d_{1}d_{2}$ indicates that both $M_{1}$ and $M_{2}$ produce defective parts.

The ergodic Markovian chain model of quality states above describes two-stage production systems having RQIF. The quality state matrix for the Markovian chain is denoted at time t.

(1) $$S_{2}(t)\;=\;{\left[P(g_{1}g_{2},t)\;P(g_{1}d_{2},t)\;P(d_{1}g_{2},t)\;P(d_{1}d_{2},t)\right]}^{T}$$

Production systems transition between the quality states based on transition probability. All of the state transition probabilities among these four states are calculated and then put into a matrix to construct the state transition probability matrix.


(2) $$C_{2}\;=\;[\begin{array}{cccc} \left(1-\alpha_{1})(1-\gamma_{2}\right) & (1-\alpha_{1})\mu_{2} & \beta_{1}(1-\eta_{2}) & \beta_{1}\theta_{2}\\ (1-\alpha_{1})\gamma_{2} & \left(1-\alpha_{1})(1-\mu_{2}\right) & \beta_{1}\eta_{2} & \beta_{1}(1-\theta_{2})\\ \alpha_{1}(1-\gamma_{2}) & \alpha_{1}\mu_{2} & \left(1-\beta_{1})(1-\eta_{2}\right) & (1-\beta_{1})\theta_{2}\\ \alpha_{1}\gamma_{2} & \alpha_{1}(1-\mu_{2}) & (1-\beta_{1})\eta_{2} & \left(1-\beta_{1})(1-\theta_{2}\right) \end{array}]$$

The final product quality is the probability by which $M_{2}$ is in a good state $g_{2}$ and produces a good product. Define $P\left(g_{2},t\right)$ as the probability of producing a product with good quality.


(3) $$P\left(g_{2},t\right)=P\left(g_{1}g_{2},t\right)+P\left(d_{1}g_{2},t\right)$$

Define the probability of producing a product with defective quality.


(4) $$P\left(d_{2},t\right)=P\left(g_{1}d_{2},t\right)+P\left(d_{1}d_{2},t\right)$$

The linear constrained equation describes the evolution of $S_{2}\left(t\right)$.


(5) $$P\left(g_{1}d_{2},t\right)+P\left(d_{1}d_{2},t\right)+P\left(d_{1}g_{2},t\right)+P\left(g_{1}g_{2},t\right)=1$$

(6) $$S_{2}\left(t+1\right)=C_{2}S_{2}\left(t\right)$$

The evolutions for $P\left(g_{2},t\right)$ and $P\left(d_{2},t\right)$ are described below.


(7) $$y_{2}\left(t\right)=\left[\begin{array}{c} P\left(g_{2},t\right)\\ P\left(d_{2},t\right) \end{array}\right]=FS_{2}\left(t\right)=\left[\begin{array}{l} \begin{array}{cccc} 1 & 0 & 1 & 0 \end{array}\\ \begin{array}{cccc} 0 & 1 & 0 & 1 \end{array} \end{array}\right]S_{2}\left(t\right)$$

The above expressions depict system transients and quality measures during transients.

## 3. Transient Quality Performance Evaluation of Two-Stage Production Systems

### 3.1. Two-Stage Production Systems with Constant Parameters

For the two-stage systems with constant parameters, the mathematical model indicates that matrix $C_{2}$ is the state transition probability matrix determined by the ergodic Markovian chain. The eigenvalues of $C_{2}$, including the unique largest eigenvalue one, can be arranged as follows.

$$1=\lambda_{1}>\lambda_{2}\geq \left|\lambda_{3}\right|\geq \left|\lambda_{4}\right|$$

According to the matrix theory, transform matrix $C_{2}$ to a diagonal matrix with non-singular matrix Z.

$$ZC_{2}Z^{-1}=diag\left[\begin{array}{cccc} 1 & \lambda_{2} & \lambda_{3} & \lambda_{4} \end{array}\right]$$

Substitute the following equation to Equations (6) and (7).


(8) $${\tilde{S}}_{2}\left(t\right)=ZS_{2}\left(t\right)$$

Transform Equations (6) and (7) as.

(9) $${\tilde{S}}_{2}(t+1)={\tilde{C}}_{2}{\tilde{S}}_{2}\left(t\right)$$

(10) $$y_{2}\left(t\right)=\tilde{F}{\tilde{S}}_{2}\left(t\right)$$

where

$${\tilde{C}}_{2}=ZC_{2}Z^{-1}=diag\left[\begin{array}{cccc} 1 & \lambda_{2} & \lambda_{3} & \lambda_{4} \end{array}\right]$$

The evolution of the system states can be calculated based on Equation (9).


(11) $${\tilde{S}}_{2}\left(t\right)={\tilde{C}}_{2}{}^{t}{\tilde{S}}_{2}\left(0\right)=diag\left[\begin{array}{cccc} 1 & \lambda_{2}^{t} & \lambda_{3}^{t} & \lambda_{4}^{t} \end{array}\right]{\tilde{S}}_{2}\left(0\right)$$

It is shown in Equation (11) that the Markovian chain of ${\tilde{S}}_{2}\left(t\right)$ reaches steady-state according to the exponential function of parameters $\lambda_{i}$ as time t evolves. With the largest eigenvalue among all four eigenvalues of matrix $C_{2}$, the second largest eigenvalue (SLE) $\lambda_{2}$ dominates the system transient duration. A large SLE approximately describes the long duration and slow convergence of the system transients.

According to Expression (10), evolutions for $P\left(d_{2},t\right)$ and $P\left(g_{2},t\right)$ are calculated.


(12) $$\left[\begin{array}{c} P\left(g_{2},t\right)\\ P\left(d_{2},t\right) \end{array}\right]=\left[\begin{array}{cccc} {\tilde{F}}_{11} & {\tilde{F}}_{12} & {\tilde{F}}_{13} & {\tilde{F}}_{14}\\ {\tilde{F}}_{21} & {\tilde{F}}_{22} & {\tilde{F}}_{23} & {\tilde{F}}_{24} \end{array}\right]diag\left[\begin{array}{cccc} 1 & \lambda_{2}^{t} & \lambda_{3}^{t} & \lambda_{4}^{t} \end{array}\right]{\tilde{S}}_{2}\left(0\right)$$

The probabilities for producing good or defective products in steady-state are denoted as $P{\left(g_{2}\right)}_{SS}$ or $P{\left(d_{2}\right)}_{SS}$, respectively.


(13) $$\begin{array}{c} P{\left(g_{2}\right)}_{SS}=\underset{t\to \infty }{\lim}P\left(g_{2},t\right)={\tilde{F}}_{11}\\ P{\left(d_{2}\right)}_{SS}=\underset{t\to \infty }{\lim}P\left(d_{2},t\right)={\tilde{F}}_{21} \end{array}$$

We have

(14) $$\left[\begin{array}{c} P\left(g_{2},t\right)\\ P\left(d_{2},t\right) \end{array}\right]=\left[\begin{array}{c} P{\left(g_{2}\right)}_{ss}\left(1+\frac{{\tilde{F}}_{12}}{{\tilde{F}}_{11}}{\tilde{S}}_{2,2}(0)\lambda_{2}^{t}+\frac{{\tilde{F}}_{13}}{{\tilde{F}}_{11}}{\tilde{S}}_{2,3}(0)\lambda_{3}^{t}+\frac{{\tilde{F}}_{14}}{{\tilde{F}}_{11}}{\tilde{S}}_{2,4}(0)\lambda_{4}^{t}\right)\\ P{\left(d_{2}\right)}_{ss}\left(1+\frac{{\tilde{F}}_{22}}{{\tilde{F}}_{21}}{\tilde{S}}_{2,2}(0)\lambda_{2}^{t}+\frac{{\tilde{F}}_{23}}{{\tilde{F}}_{21}}{\tilde{S}}_{2,3}(0)\lambda_{3}^{t}+\frac{{\tilde{F}}_{24}}{{\tilde{F}}_{21}}{\tilde{S}}_{2,4}(0)\lambda_{4}^{t}\right) \end{array}\right]$$

It is shown in Equation (14) that transients of system quality $P\left(g_{2},t\right)$ and $P\left(d_{2},t\right)$ are described by both eigenvalues $\lambda_{i}$ of the transition probability matrix $C_{2}$ and pre-exponential factors (PEFs). $\frac{{\tilde{F}}_{ij}}{{\tilde{F}}_{i1}}$. Corresponding to the SLE $\lambda_{2}$, the PEFs $\frac{{\tilde{F}}_{12}}{{\tilde{F}}_{11}}$ and $\frac{{\tilde{F}}_{22}}{{\tilde{F}}_{21}}$ are most important.


(15) $$\Phi_{1}=\left|\frac{{\tilde{F}}_{12}}{{\tilde{F}}_{11}}\right|,\;\Phi_{1}=\left|\frac{{\tilde{F}}_{22}}{{\tilde{F}}_{21}}\right|$$

Factors of $\Phi_{1}$, $\Phi_{2}$ describe the extent of the effects of SLE on product quality transients. Large factors describe large effects and, thus, slow transients.

### 3.2. Two-Stage Production Systems with Time-Varying Parameters

When the parameters of a production system change over time, the system is described using the inhomogeneous Markovian chain. Assumption 6 in Section 2.1 should be modified to incorporate system properties of time-varying parameters. Let $\alpha_{1}\left(t\right),\gamma_{2}\left(t\right),\eta_{2}\left(t\right)$ and $\beta_{1}\left(t\right),\mu_{2}\left(t\right),\theta_{2}\left(t\right)$ denote system quality repair and failure probability. State transition probability matrix $V_{2}\left(t\right)$ of this Markovian chain at time t can be calculated.


(16) $$V_{2}\left(t\right)=\left[\begin{array}{cccc} \left(1-\alpha_{1}\left(t\right)\right)\left(1-\gamma_{2}\left(t\right)\right) & \left(1-\alpha_{1}\left(t\right)\right)\mu_{2}\left(t\right) & \beta_{1}\left(t\right)\left(1-\eta_{2}\left(t\right)\right) & \beta_{1}\left(t\right)\theta_{2}\left(t\right)\\ \left(1-\alpha_{1}\left(t\right)\right)\gamma_{2}\left(t\right) & \left(1-\alpha_{1}\left(t\right)\right)\left(1-\mu_{2}\left(t\right)\right) & \beta_{1}\left(t\right)\eta_{2}\left(t\right) & \beta_{1}\left(t\right)\left(1-\theta_{2}\left(t\right)\right)\\ \alpha_{1}\left(t\right)\left(1-\gamma_{2}\left(t\right)\right) & \alpha_{1}\left(t\right)\mu_{2}\left(t\right) & \left(1-\beta_{1}\left(t\right)\right)\left(1-\eta_{2}\left(t\right)\right) & \left(1-\beta_{1}\left(t\right)\right)\theta_{2}\left(t\right)\\ \alpha_{1}\left(t\right)\gamma_{2}\left(t\right) & \alpha_{1}\left(t\right)\left(1-\mu_{2}\left(t\right)\right) & \left(1-\beta_{1}\left(t\right)\right)\eta_{2}\left(t\right) & \left(1-\beta_{1}\left(t\right)\right)\left(1-\theta_{2}\left(t\right)\right) \end{array}\right]$$

The evolutions for system state $S_{2}\left(t\right)$ are given by.


(17) $$P\left(g_{1}g_{2},t\right)+P\left(d_{1}d_{2},t\right)+P\left(g_{1}d_{2},t\right)+P\left(d_{1}g_{2},t\right)=1$$

(18) $$S_{2}\left(t+1\right)=V_{2}\left(t\right)S_{2}\left(t\right)$$

Considering the substitution,

(19) $$\begin{array}{c} Z\left(t\right)V_{2}\left(t\right)Z^{-1}\left(t\right)=diag\left[\begin{array}{cccc} 1 & \lambda_{2}\left(t\right) & \lambda_{3}\left(t\right) & \lambda_{4}\left(t\right) \end{array}\right]\\ {\tilde{S}}_{2}\left(t\right)=Z\left(t\right)S_{2}\left(t\right) \end{array}$$

it follows that

(20) $${\tilde{S}}_{2}\left(t+1\right)={\tilde{V}}_{2}\left(t\right){\tilde{S}}_{2}\left(t\right)$$

(21) $$y_{2}\left(t\right)=\tilde{F}\left(t\right){\tilde{S}}_{2}\left(t\right)$$

The evolution for state ${\tilde{S}}_{2}\left(t\right)$ is expressed by


(22) $${\tilde{S}}_{2}\left(t\right)=\prod_{k=0}^{t}{\tilde{V}}_{2}\left(k\right){\tilde{S}}_{2}\left(0\right)=diag\left[\begin{array}{cccc} 1 & \prod_{k=0}^{t}\lambda_{2}\left(k\right) & \prod_{k=0}^{t}\lambda_{3}\left(k\right) & \prod_{k=0}^{t}\lambda_{4}\left(k\right) \end{array}\right]{\tilde{S}}_{2}\left(0\right)$$

According to Expression (21), the evolutions for $P\left(g_{2},t\right)$ and $P\left(d_{2},t\right)$ are calculated.


(23) $$\left[\begin{array}{c} P\left(g_{2},t\right)\\ P\left(d_{2},t\right) \end{array}\right]=\left[\begin{array}{c} P{\left(g_{2}\right)}_{ss}\left(1+\frac{{\tilde{F}}_{12}}{{\tilde{F}}_{11}}{\tilde{S}}_{2,2}(0)\prod_{k=0}^{t}\lambda_{2}\left(k\right)+\frac{{\tilde{F}}_{13}}{{\tilde{F}}_{11}}{\tilde{S}}_{2,3}(0)\prod_{k=0}^{t}\lambda_{3}\left(k\right)+\frac{{\tilde{F}}_{14}}{{\tilde{F}}_{11}}{\tilde{S}}_{2,4}(0)\prod_{k=0}^{t}\lambda_{4}\left(k\right)\right)\\ P{\left(d_{2}\right)}_{ss}\left(1+\frac{{\tilde{F}}_{22}}{{\tilde{F}}_{21}}{\tilde{S}}_{2,2}(0)\prod_{k=0}^{t}\lambda_{2}\left(k\right)+\frac{{\tilde{F}}_{23}}{{\tilde{F}}_{21}}{\tilde{S}}_{2,3}(0)\prod_{k=0}^{t}\lambda_{3}\left(k\right)+\frac{{\tilde{F}}_{24}}{{\tilde{F}}_{21}}{\tilde{S}}_{2,4}(0)\prod_{k=0}^{t}\lambda_{4}\left(k\right)\right) \end{array}\right]$$

In the following section, we will use the results of the two-stage systems with time-varying parameters to describe the transient quality behavior for multi-stage production systems.

## 4. Transient Quality Performance Evaluation of Multi-Stage Production Systems

### 4.1. Aggregation-Based Approach for Multi-Stage Systems

To generally establish the quality propagation model of multi-stage production systems during transients, consider the three-stage system and bring in the idea of the equivalent aggregation technique. In the three-stage system, calculate the probability by which $M_{3}$ produces a good product. The product quality of $M_{3}$ is characterized by both the current state in $M_{3}$ and the incoming product quality from the upstream stage $M_{2}$. Moreover, the output quality in stage $M_{2}$ is equivalent with the final product quality of the two-stage system $M_{1}-M_{2}$. The quality of system $M_{1}-M_{2}$ can be calculated in Section 3. It is possible to construct a single virtual stage $M_{2}^{\prime }$ to represent the aggregated quality behavior of the two-stage system, $M_{1}-M_{2}$. In other words, we can view the incoming parts for stage $M_{3}$ as processed by a modified version of $M_{2}$ with consideration of the effects of both $M_{1}$ and $M_{2}$. Thus, the approach to calculate final quality of the three-stage system is depicted as follows. Firstly, merge stages $M_{1}$ and $M_{2}$ to a merged stage $M_{2}^{\prime }$. Then construct the model of the new two-stage system $M_{2}^{\prime }-M_{3}$ and calculate the final product quality using the method for the two-stage systems described above.

Next, we will obtain the parameters of the virtual stage $M_{2}^{\prime }$. For the two-stage system $M_{2}^{\prime }-M_{3}$; the system has six transition probability parameters. $\gamma_{3},\eta_{3},\mu_{3},\theta_{3}$ are the parameters of stage $M_{3}$, $\alpha_{2}^{\prime }\left(t\right)$ and $\beta_{2}^{\prime }\left(t\right)$ are the parameters of the merged stage $M_{2}^{\prime }$. The quality failure probability $\alpha_{2}^{\prime }\left(t\right)$ defines the probability of $M_{2}^{\prime }$ transiting from the good state $g_{2}^{\prime }$ to the defective state $d_{2}^{\prime }$. Thus, it equals with probability of the two-stage system $M_{1}-M_{2}$, transiting from the states $g_{1}g_{2}$ or $d_{1}g_{2}$ to the states $g_{1}d_{2}$ or $d_{1}d_{2}$ during the time slot t. It follows that:

$$\alpha_{2}^{\prime }\left(t\right)=\frac{P\left(g_{1}g_{2},t\right)\gamma_{2}+P\left(d_{1}g_{2},t\right)\eta_{2}}{P\left(g_{1}g_{2},t\right)+P\left(d_{1}g_{2},t\right)}$$

Similarly,

$$\beta_{2}^{\prime }\left(t\right)=\frac{P\left(g_{1}d_{2},t\right)\mu_{2}+P\left(d_{1}d_{2},t\right)\theta_{2}}{P\left(g_{1}d_{2},t\right)+P\left(d_{1}d_{2},t\right)}$$

With the quality repair and failure probability of $M_{2}^{\prime }$ calculated, we are able to calculate the transient quality performance. Define $P\left(g_{3},t\right)$ as the probability of producing a good product in a three-stage system.

$$P\left(g_{3},t\right)=P\left(g_{2}g_{3},t\right)+P\left(d_{2}g_{3},t\right)$$

**Figure 3:**
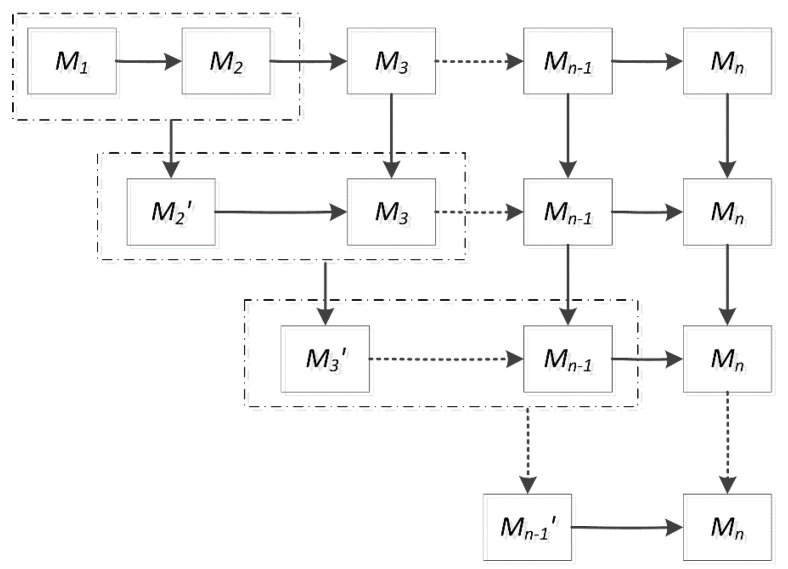
The recursive process for multi-stage production systems.

The general recursive process for a multi-stage production system is described in Figure 3. The final quality for a multi-stage system is obtained by conducting iteration procedures and solving a series of the two-stage system. Using the Markovian model developed in Section 3, the quality of the two-stage system $M_{1}-M_{2}$ is obtained. Moreover, stages $M_{1}$ and $M_{2}$ are aggregated to the aggregated stage $M_{2}^{\prime }$. Establish the system quality of the model for the new two-stage system $M_{2}^{\prime }-M_{3}$, after that, stages $M_{2}^{\prime }$ and $M_{3}$ are aggregated to the aggregated stage $M_{3}^{\prime }$. Carry out the recursive procedures, and the previous $\left(n-1\right)$ stages are aggregated to the aggregated stage $M_{n-1}^{\prime }$. Finally, establish the product quality of the model for the last two-stage system $M_{n-1}^{\prime }-M_{n}$.

Six fundamental system parameters, in total, for any two-stage system $M_{i}^{\prime }-M_{i+1}$ exists. Parameters $\gamma_{i+1},\eta_{i+1},\mu_{i+1},\theta_{i+1}$ reflect the characteristics for stage $M_{i+1}$. Parameters $\alpha_{i}^{\prime }\left(t\right),\beta_{i}^{\prime }\left(t\right)$ reflect the characteristics for the new aggregated stage $M_{i}^{\prime }$.


(24) $$\begin{array}{cc} \alpha_{i}^{\prime }\left(t\right)= & \mathrm{Prob}[\left.M_{i}^{\prime }\;\;\mathrm{in}\;\mathrm{defective}\;\mathrm{state}\;\mathrm{at}\;\mathrm{time}\;\mathrm{slot}\;t+1\;\right|\;M_{i}^{\prime }\;\mathrm{in}\;\mathrm{good}\;\mathrm{state}\;\mathrm{at}\;\mathrm{time}\;\mathrm{slot}\;t]\\ = & \mathrm{Prob}[\left.i\mathrm{th}\;\mathrm{stage}\;\mathrm{produces}\;a\;\mathrm{defective}\;\mathrm{part}\;\mathrm{at}\;\mathrm{time}\;\mathrm{slot}\;t+1\;\right|\;\\ & \;i\mathrm{th}\;\mathrm{stage}\;\mathrm{produces}\;a\;\mathrm{good}\;\mathrm{part}\;\mathrm{at}\;\mathrm{time}\;\mathrm{slot}\;t]\\ = & \frac{P\left(d_{i-1}g_{i},t\right)\eta_{i}+P\left(g_{i-1}g_{i},t\right)\gamma_{i}}{P\left(d_{i-1}g_{i},t\right)+P\left(g_{i-1}g_{i},t\right)} \end{array}$$

(25) $$\begin{array}{cc} \beta_{i}^{\prime }\left(t\right)= & \mathrm{Prob}[\left.M_{i}^{\prime }\;\mathrm{in}\;\mathrm{good}\;\mathrm{state}\;\mathrm{at}\;\mathrm{time}\;\mathrm{slot}\;t+1\;\right|\;M_{i}^{\prime }\;\mathrm{in}\;\mathrm{defective}\;\mathrm{state}\;\mathrm{at}\;\mathrm{time}\;\mathrm{slot}\;t]\\ = & \mathrm{Prob}[\left.i\mathrm{th}\;\mathrm{stage}\;\mathrm{produces}\;a\;\mathrm{good}\;\mathrm{part}\;\;\mathrm{at}\;\mathrm{time}\;\mathrm{slot}\;t+1\;\right|\;\\ & i\mathrm{th}\;\mathrm{stage}\;\mathrm{produces}\;a\;\mathrm{defective}\;\mathrm{part}\;\mathrm{at}\;\mathrm{time}\;\mathrm{slot}\;t]\\ = & \frac{P\left(d_{i-1}d_{i},t\right)\theta_{i}+P\left(g_{i-1}d_{i},t\right)\mu_{i}}{P\left(d_{i-1}d_{i},t\right)+P\left(g_{i-1}d_{i},t\right)} \end{array}$$

Secondly, put the state transition probability into the matrix to establish the transition probability matrix.

(26) $$C_{i+1}\left(t\right)=\left[\begin{array}{cccc} \left(1-\alpha_{i}^{\prime }\left(t\right)\right)\left(1-\gamma_{i+1}\right) & \left(1-\alpha_{i}^{\prime }\left(t\right)\right)\mu_{i+1} & \beta_{i}^{\prime }\left(t\right)\left(1-\eta_{i+1}\right) & \beta_{i}^{\prime }\left(t\right)\theta_{i+1}\\ \left(1-\alpha_{i}^{\prime }\left(t\right)\right)\gamma_{i+1} & \left(1-\alpha_{i}^{\prime }\left(t\right)\right)\left(1-\mu_{i+1}\right) & \beta_{i}^{\prime }\left(t\right)\eta_{i+1} & \beta_{i}^{\prime }\left(t\right)\left(1-\theta_{i+1}\right)\\ \alpha_{i}^{\prime }\left(t\right)\left(1-\gamma_{i+1}\right) & \alpha_{i}^{\prime }\left(t\right)\mu_{i+1} & \left(1-\beta_{i}^{\prime }\left(t\right)\right)\left(1-\eta_{i+1}\right) & \left(1-\beta_{i}^{\prime }\left(t\right)\right)\theta_{i+1}\\ \alpha_{i}^{\prime }\left(t\right)\gamma_{i+1} & \alpha_{i}^{\prime }\left(t\right)\left(1-\mu_{i+1}\right) & \left(1-\beta_{i}^{\prime }\left(t\right)\right)\eta_{i+1} & \left(1-\beta_{i}^{\prime }\left(t\right)\right)\left(1-\theta_{i+1}\right) \end{array}\right]$$

The system states matrix at a certain time can be defined.


(27) $$S_{i+1}\left(t\right)={\left[\begin{array}{cccc} P\left(g_{i}g_{i+1},t\right) & P\left(g_{i}d_{i+1},t\right) & P\left(d_{i}g_{i+1},t\right) & P\left(d_{i}d_{i+1},t\right) \end{array}\right]}^{T}$$

The evolution for $S_{i+1}\left(t\right)$ is depicted by the following linear equations.


(28) $$\begin{array}{c} P\left(g_{i}g_{i+1},t\right)+P\left(g_{i}d_{i+1},t\right)+P\left(d_{i}g_{i+1},t\right)+P\left(d_{i}d_{i+1},t\right)=1\\ S_{i+1}\left(t+1\right)=C_{i+1}\left(t\right)S_{i+1}\left(t\right) \end{array}$$

The product quality of stage $M_{i+1}$ through the multi-stage production system is the probability by which $M_{i+1}$ is in state $g_{i+1}$ to produce a good product. Define $P\left(g_{i+1},t\right)$ as the probability to produce a good product in stage $M_{i+1}$ through the system.


(29) $$P\left(g_{i+1},t\right)=P\left(d_{i}g_{i+1},t\right)+P\left(g_{i}g_{i+1},t\right)$$

Define $P\left(g_{n},t\right)$ as the final product quality for a multi-stage production system.


(30) $$P\left(g_{n},t\right)=P\left(g_{n-1}g_{n},t\right)+P\left(d_{n-1}g_{n},t\right)$$

**Figure 4:**
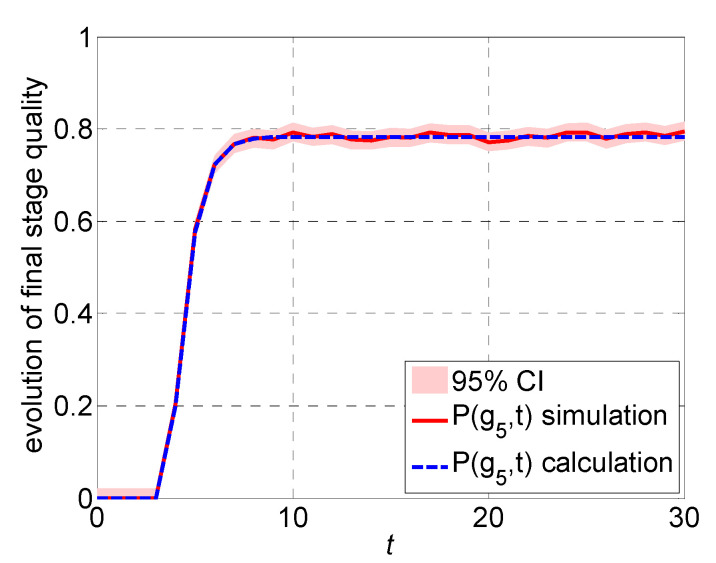
The evolution of product quality: comparison between calculation and simulation with 95% confidence interval.

Consider a five-stage production system with the following quality repair and failure probabilities; $\alpha_{1}=0.1$, $\beta_{1}=0.8$, $\gamma_{i}=0.1$, $\eta_{i}=0.5$, $\mu_{i}=0.8$, $\theta_{i}=0.2$, i=2,3,4,5. The evolution of product quality with a comparison between calculation and simulation is presented in Figure 4. The solid line depicts the simulated performance while the shaded region indicates 95% confidence interval. The dashed line depicts the calculation using the analytical method derived. The simulation result and analytical calculation are close during the entire production time. The calculated product quality can clearly track system dynamics during transients, which illustrates the effectiveness of the transient quality analysis of multi-stage production systems.

### 4.2. Model Accuracy Investigation

To quantitatively evaluate the accuracy of the derived methods, comparisons are made between the approximate analytical calculation and simulation results. Simulation parameters are selected equiprobably and randomly from the pre-defined value range sets. Regarding every setting of the parameters, one thousand replications were conducted for each simulation. The experiment process for each setting of parameters is presented.

1. Generate a setting of system parameters equiprobably and randomly among the following value sets.
  (1) The size–number of stages belong to $\left[2,10\right]$.
  (2) The quality failure probability in case of an incoming product with good quality has a relatively small value, i.e., $\alpha_{1}\in \left[0,0.1\right]$, $\gamma_{i}\in \left[0,0.1\right]$.
  (3) The quality repair probability in case of an incoming product with good quality has a relatively large value, i.e., $\beta_{1}\in \left[0.6,0.9\right]$, $\mu_{i}\in \left[0.6,0.9\right]$.
  (4) The quality repair probability and failure probability in case of an incoming product with a defective quality, $\eta_{i}\in \left[0,0.6\right]$ and $\theta_{i}\in \left[0,0.4\right]$.
2. Conduct simulations for 200 time slots.
3. Quality performance $P\left(g_{n},t\right)$ is unknown. Since the simulated performance measure is unbiased, the performance measure in the simulation is utilized for reflecting $P\left(g_{n},t\right)$.
4. Calculate the average value for performance measure during last 100 time slots. Moreover, the average is denoted as the simulated value of the steady-state quality.

$$\tilde{P}{\left(g_{n}\right)}_{SS}=\frac{1}{100}\sum_{t=T-99}^{T}\tilde{P}(g_{n},t)$$

The error metric to investigate the accuracy is denoted as:

$$\delta_{P(g_{n})}=\frac{1}{T}\sum_{t=1}^{T}\frac{\left|\tilde{P}(g_{n},t)-P(g_{n},t)\right|}{\tilde{P}{\left(g_{n}\right)}_{SS}}\times 100\%$$

**Figure 5:**
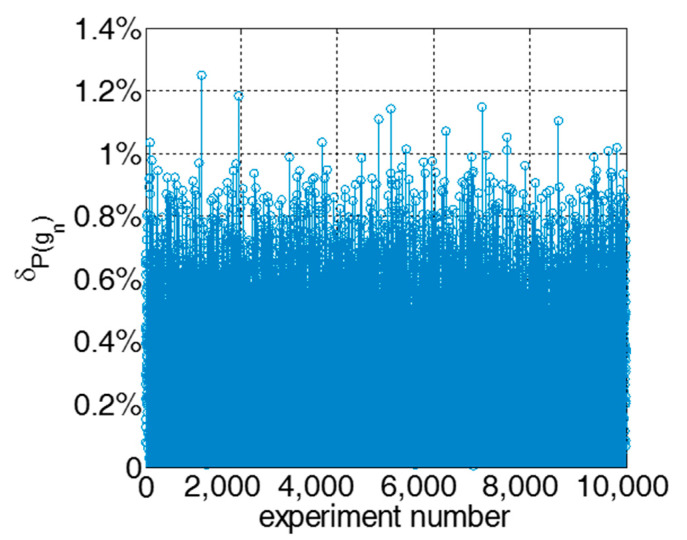
Difference of the system quality performance between the analytical model and simulation.

A total of 10,000 parameter settings randomly generated were investigated using both simulation and analytical models. The accuracy results for each experiment are presented in Figure 5. The quality performance measure calculated by analytical methods have small errors and are rather close to the simulation results. Specifically, the mean error of $\delta_{P\left(g_{n}\right)}$ is 0.57%. The maximum value of $\delta_{P\left(g_{n}\right)}$ among the 10,000 experiments is 1.26%. The analytical model and the aggregation-based iterative procedure can deliver high accuracy in a transient quality analysis of a multi-stage production system.

## 5. Analysis of the System Theoretic Properties

### 5.1. Analysis of Settling Time

The settling time defines the time necessary of a system quality $P\left(g_{n},t\right)$ approaching and maintaining in ±3% ranges of the steady-state values.


(31) $$t_{S}=\inf\left\{t\left|\frac{\left|P(g_{n},t)-P{\left(g_{n}\right)}_{SS}\right|}{P{\left(g_{n}\right)}_{SS}}\right.<=3\%\right\}$$

To justify the accuracy of the settling time (31), we conducted a numerical analysis by randomly selecting system parameters from range sets in Section 4.2. The $t_{S}$ is solved using an analytical calculation and $\hat{t_{S}}$ is solved using a simulation. The accuracy is quantitatively evaluated.

(32) $$\delta_{t_{S}}=\left|t_{S}-\hat{t_{S}}\right|$$

In approximately 89% of all cases investigated, the calculated $t_{S}$ is within two time slots from the simulated value $\hat{t_{S}}$, which proves the accuracy of the analytical calculation.

Under assumptions 1–6, settling time $t_{S}$ is the function of system parameters, quality failure probabilities $\alpha_{1},\gamma_{i},\eta_{i}$, and quality repair probabilities $\beta_{1},\mu_{i},\theta_{i}$, as well as the number of stages n. To investigate properties of $t_{S}$, extensive numerical experiments are implemented through selections of the system parameters equiprobably and randomly from value range sets. For simplicity, consider the cases in which transition probability parameters are identical of each stage, with good and defective incoming products, respectively, defined as equal stage cases.

(33) $$\gamma_{i}=\alpha_{1},\mu_{i}=\beta_{1},\eta_{i}=\eta_{2},\theta_{i}=\theta_{2}$$

We firstly explore property of $t_{S}$ in terms of $\alpha_{1}$ and $\beta_{1}$ and then in terms of $\eta_{2}$ and $\theta_{2}$.

**Figure 6:**
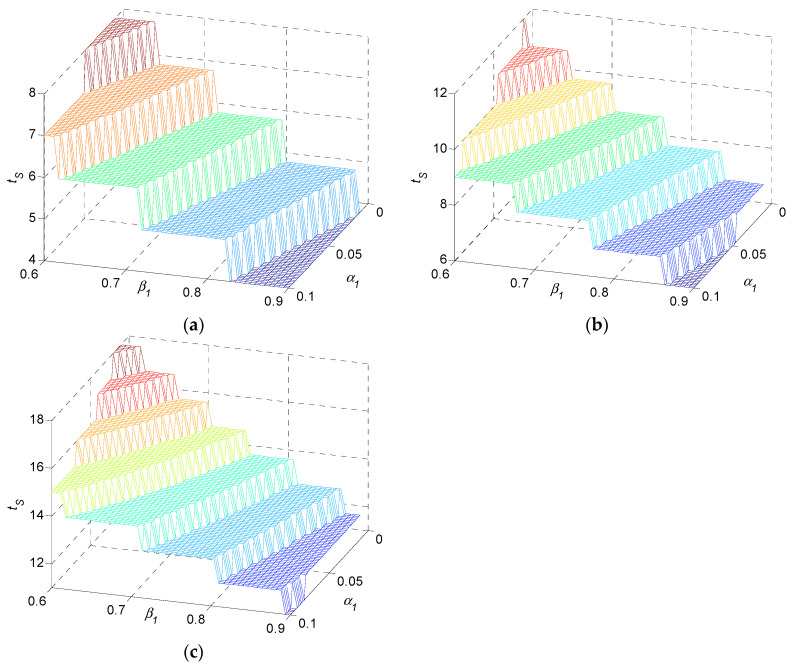
Settling time as a function of $\alpha_{1}$ and $\beta_{1}$ for (**a**) three-stage, (**b**) five-stage, and (**c**) ten-stage production systems.

In terms of $\alpha_{1}$ and $\beta_{1}$, three examples are typically presented in Figure 6 due to space limits, instead of presenting all multi-stage production systems investigated extensively. Examples are (a) three-stage systems, (b) five-stage systems, (c) ten-stage systems. The monotonic property of $t_{S}$ regarding system parameters $\alpha_{1}$ and $\beta_{1}$ is presented for three examples while $\eta_{2}=0.5$, $\theta_{2}=0.2$. As illustrated in the figure, settling time $t_{S}$ decreases in $\alpha_{1}$ and decreases in $\beta_{1}$. It increases in n.

Numerical result 1: settling time $t_{S}$ in multi-stage production systems is a decreasing function in $\alpha_{1}$ and $\beta_{1}$. It is an increasing function of n.

Remark 1: it should be noted that the three examples of Figure 6 are shown as illustrations. In fact, numerical result 1 can be observed on a general basis in a multi-stage production system under the consideration of assumptions 1–6, which is not only in the examples illustrated. This remark also applies regarding numerical results 2 to 5.

**Figure 7:**
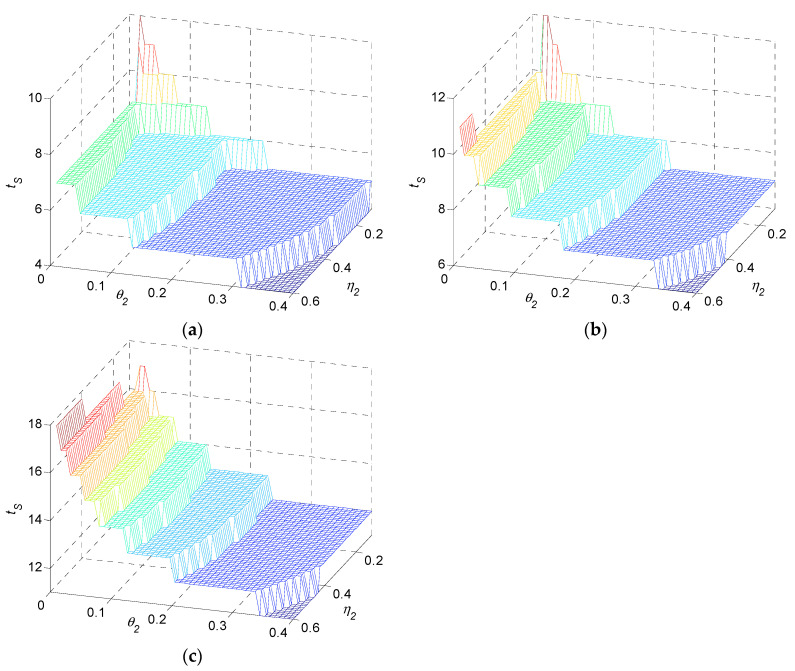
Settling time as functions of $\eta_{2}$ and $\theta_{2}$ for (**a**) three-stage, (**b**) five-stage, and (**c**) ten-stage production systems.

Similarly, in terms of $\eta_{2}$ and $\theta_{2}$, three typical example systems are presented in Figure 7, while $\alpha_{1}=0.1$, $\beta_{1}=0.8$. $t_{S}$ decreases in $\eta_{2}$ and decreases in $\theta_{2}$. It increases in n.

Numerical result 2: settling time $t_{S}$ in a multi-stage production system is a decreasing function in $\eta_{2}$ and $\theta_{2}$. It is an increasing function of n.

Remark 2: as shown in numerical results 1 and 2, if $\alpha_{1}$, $\beta_{1}$, $\eta_{2}$ or $\theta_{2}$ increases, the settling time in the multi-stage production system is generally reduced, which leads to a shorter transient duration. With the number of stages n increasing, the system suffers slower convergence. As the direct metric of the system transient duration, settling time is actually the joint impact for system properties, including the SLE and PEF, on quality transients.

### 5.2. Analysis of Quality Loss

The initial conditions of the production systems have significant effects upon system quality transients. In a fresh restart production after product changes or preventive maintenance, the production system usually operates in a defective quality state determined by frequent fixture relocating errors. Typically system quality converges towards steady-state from below its steady-state measure, leading to system quality loss during transients.

The actual system quality for T time slots is the integration of system quality performance $P\left(g_{n},t\right)$ from 0 to T. The expected system quality is the integration of steady-state quality $P{\left(g_{n}\right)}_{SS}$. Quality loss of multi-stage production systems for a period T is defined as.


(34) $$L_{Q}(S_{n}(0))=\sum_{t=0}^{T}\left[P{\left(g_{n}\right)}_{SS}-P(g_{n},t;S_{n}(0))\right]$$

Define $QLR\left(t\right)$ as the quality loss rate, which is the percentage of quality loss compared with steady-state over time t.


(35) $$QLR\left(t\right)=\frac{L_{Q}(S_{n}(0),t)}{t\times P{\left(g_{n}\right)}_{SS}}\times 100\%$$

**Figure 8:**
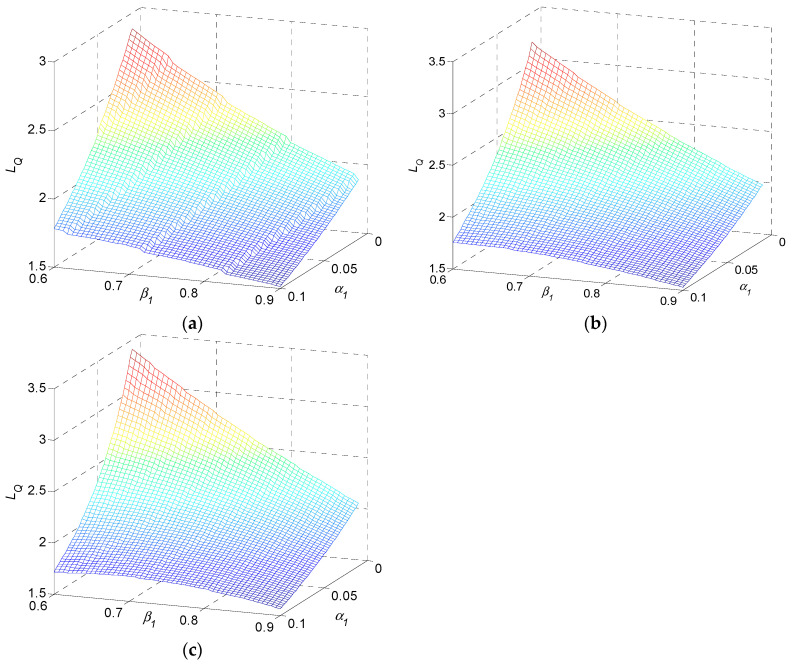
Quality loss as a function of $\alpha_{1}$ and $\beta_{1}$ for (**a**) three-stage, (**b**) five-stage, and (**c**) ten-stage production systems.

In terms of $\alpha_{1}$ and $\beta_{1}$, the monotonic property of quality loss is presented as in Figure 8 for the examples, while $\eta_{2}=0.5$, $\theta_{2}=0.2$. Quality loss monotonically decreases in $\alpha_{1}$ and decreases in $\beta_{1}$. It increases in n.

Numerical result 3: the quality loss of a multi-stage production system during transients monotonically decreases in $\alpha_{1}$ and $\beta_{1}$. It increases in n.

**Figure 9:**
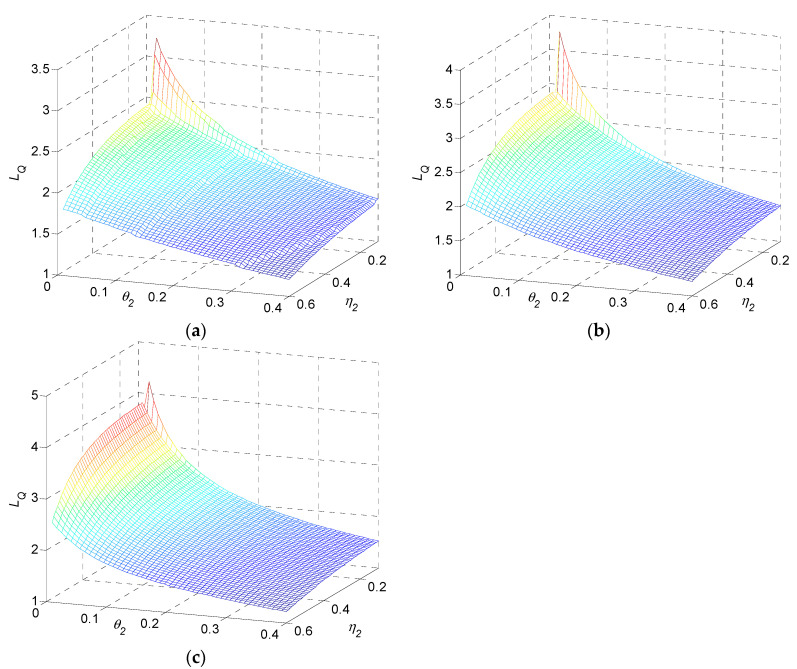
Quality loss as a function of $\eta_{2}$ and $\theta_{2}$ for (**a**) three-stage, (**b**) five-stage, and (**c**) ten-stage production systems.

In terms of $\eta_{2}$ and $\theta_{2}$, monotonic property of quality loss is presented as in Figure 9. Quality loss monotonically decreases in $\eta_{2}$ and decreases in $\theta_{2}$. It increases in n.

Numerical result 4: quality loss of a multi-stage production system during transients monotonically decreases in $\eta_{2}$ and $\theta_{2}$, and increases in n.

Remark 3: as shown in numerical results 3 and 4, if $\alpha_{1}$, $\beta_{1}$, $\eta_{2}$, or $\theta_{2}$ increases, quality loss during transients is practically reduced. The settling time and quality loss shown in the figures indicate that quality loss $L_{Q}$ has a strong relationship with settling time $t_{S}$. A long duration of transients results in a large quality loss, in general.

### 5.3. Steady-State Quality and Continuous Improvement Analysis

**Figure 10:**
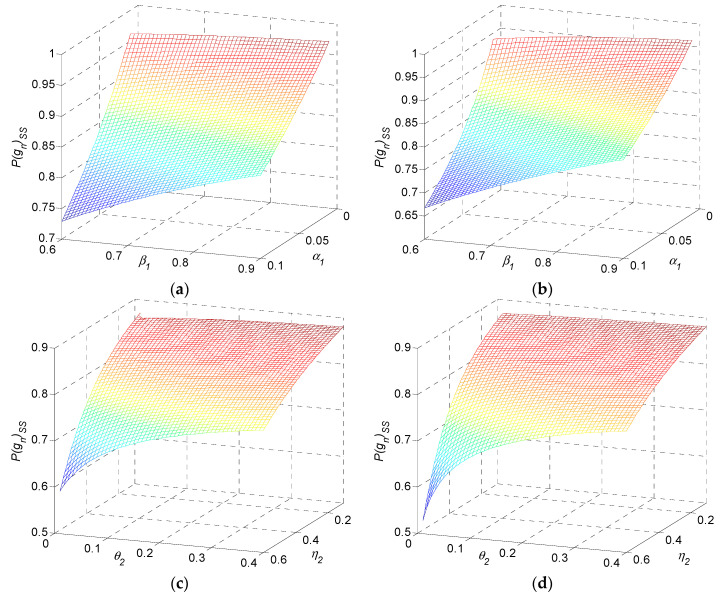
Steady-state qualities as functions of $\alpha_{1}$ and $\beta_{1}$ for (**a**) three-stage and (**b**) ten-stage systems, as functions of $\eta_{2}$ and $\theta_{2}$ for (**c**) three-stage and (**d**) ten-stage systems.

In this subsection, we show that quality performance of steady-state operates in a different manner from the transient phase. Quality performance still needs to be comprehensively explored in both transient and steady-state frameworks to provide directions, to plan continuous improvements. As an illustration, the monotonic property of steady-state quality with respect to parameter $\alpha_{1}$ and $\beta_{1}$ is presented in Figure 10a,b; $\eta_{2}=0.5$, $\theta_{2}=0.2$. Steady-state quality monotonically decreases in $\alpha_{1}$ and increases in $\beta_{1}$. The monotonic property for steady-state quality regarding parameter $\eta_{2}$ and $\theta_{2}$ is presented in Figure 10c,d; $\alpha_{1}=0.1$, $\beta_{1}=0.8$. Steady-state quality monotonically decreases in $\eta_{2}$ and increases in $\theta_{2}$.

Numerical result 5: steady-state quality of a multi-stage production system monotonically decreases in $\alpha_{1}$ and $\eta_{2}$ and increases in $\beta_{1}$ and $\theta_{2}$. The monotonicity property—that the steady-state quality is expected to be a decreasing function of the number of stages n—may not hold.

Remark 4: intuitively, we may expect that the final quality will decline as the size of the production system increases. However, such properties may not hold in multi-stage production systems with RQIF. As shown in Figure 10a,b, steady-state quality only shows a slight decrease for a wide range of ($\alpha_{1}$, $\beta_{1}$) as the number n increases from three-stage to ten-stage. It shows a steep decline when $\alpha_{1}$ is large and $\beta_{1}$ is small, at the same time. In Figure 10c,d, this property also applies in case of defective incoming products. This phenomenon is just a representation of the characteristics of quality propagation of multi-stage production systems with RQIF. In such systems, product quality depends on both the incoming product quality and the states of stages. Incoming products have defective or good qualities before processed at each stage. Both quality corrections and quality degradations exist in production systems. A defective product may be corrected by the downstream stage. Thus, the final quality does not necessarily decline in a longer production line. Only in case of small quality repair probability and large failure probability of long production lines will the steady-state quality performance drop sharply. For quality improvement, we should avoid the situation where large quality failure probability and small repair probability occur simultaneously in a multi-stage production system.

Remark 5: the impacts of quality failure probabilities $\alpha_{1}$ and $\eta_{2}$ on transient quality performance are qualitatively different from those on steady-state quality. Increase of $\alpha_{1}$ and $\eta_{2}$ can reduce quality loss during transients; however, it impedes quality in the steady-state. On the other hand, an increase of $\beta_{1}$ and $\theta_{2}$ can facilitate both quality loss reduction and steady-state quality improvement. It is more favorable to improve $\beta_{1}$ and $\theta_{2}$ than $\alpha_{1}$ and $\eta_{2}$ for continuous improvement, providing practical guidance for operation management to achieve better quality.

### 5.4. Bottleneck Analysis

The final product quality is influenced by the variations accumulatively introduced and propagated as the product moves along a multi-stage system. To improve the quality more effectively, attention should be placed on the system parameter whose change will result in the largest improvement of quality performance. The one certain stage or certain transition parameter that impedes quality performance to the strongest extent is the denoted quality bottleneck.

In context of quality loss during transients, denote the quality bottleneck stage as in which stage quality loss possibly undergoes the largest increase. The quality loss in the i stage of a multi-stage production system is defined as.


(36) $$L_{Q}(S_{i}(0))=\sum_{t=0}^{T}\left[P{\left(g_{i}\right)}_{SS}-P(g_{i},t;S_{i}(0))\right]$$

Denote the quality loss change after stage $M_{i}$ as


(37) $$\Delta L_{Q}(S_{i}(0))=L_{Q}(S_{i}(0))-L_{Q}(S_{i-1}(0))$$

The quality bottleneck stage will be the one with the largest positive value of quality loss change.


(38) $$\max\left\{\Delta L_{Q}(S_{i}(0))\right\}$$

In the next step, we will change a parameter in the bottleneck stage and see which one brings in the maximum benefit to quality improvement of $L_{Q}\left(S_{i}\left(0\right)\right)$. The special parameter that impedes $L_{Q}\left(S_{i}\left(0\right)\right)$ to the greatest extent is the quality bottleneck parameter. This process can be regarded as a sensitivity analysis for $L_{Q}\left(S_{i}\left(0\right)\right)$ regarding parameter $\gamma_{i},\eta_{i},\mu_{i},\theta_{i}$ of stage $M_{i}$. Change only a parameter at one time while the other parameters remain unchanged. Correspondingly, denote the changed parameters as $\gamma_{i}^{\prime },\eta_{i}^{\prime },\mu_{i}^{\prime },\theta_{i}^{\prime }$, and denote the changed quality loss in stage $M_{i}$ as $L_{Q}\left(S_{i}\left(0\right),\gamma_{i}^{\prime }\right)$, $L_{Q}\left(S_{i}\left(0\right),\eta_{i}^{\prime }\right)$, $L_{Q}\left(S_{i}\left(0\right),\mu_{i}^{\prime }\right)$, $L_{Q}\left(S_{i}\left(0\right),\theta_{i}^{\prime }\right)$. The quality bottleneck parameter for stage $M_{i}$ regarding $\gamma_{i}$ is the QBN-$\gamma_{i}$ formulated as

(39) $$\frac{\left|L_{Q}\left(S_{i}\left(0\right),\gamma_{i}^{\prime }\right)-L_{Q}\left(S_{i}\left(0\right)\right)\right|/L_{Q}\left(S_{i}\left(0\right)\right)}{\left|\gamma_{i}^{\prime }-\gamma_{i}\right|/\gamma_{i}}$$

Similarly, the quality bottleneck parameter for stage $M_{i}$ regarding $\eta_{i}$ is the QBN-$\eta_{i}$, formulated as

(40) $$\frac{\left|L_{Q}\left(S_{i}\left(0\right),\eta_{i}^{\prime }\right)-L_{Q}\left(S_{i}\left(0\right)\right)\right|/L_{Q}\left(S_{i}\left(0\right)\right)}{\left|\eta_{i}^{\prime }-\eta_{i}\right|/\eta_{i}}$$

The quality bottleneck parameter for stage $M_{i}$, regarding $\mu_{i}$, is the QBN-$\mu_{i}$, formulated as

(41) $$\frac{\left|L_{Q}\left(S_{i}\left(0\right),\mu_{i}^{\prime }\right)-L_{Q}\left(S_{i}\left(0\right)\right)\right|/L_{Q}\left(S_{i}\left(0\right)\right)}{\left|\mu_{i}^{\prime }-\mu_{i}\right|/\mu_{i}}$$

The quality bottleneck parameter for stage $M_{i}$, regarding $\theta_{i}$, is the QBN-$\theta_{i}$, formulated as

(42) $$\frac{\left|L_{Q}\left(S_{i}\left(0\right),\theta_{i}^{\prime }\right)-L_{Q}\left(S_{i}\left(0\right)\right)\right|/L_{Q}\left(S_{i}\left(0\right)\right)}{\left|\theta_{i}^{\prime }-\theta_{i}\right|/\theta_{i}}$$

The above quality bottleneck parameters QBN-$\gamma_{i}$, QBN-$\eta_{i}$, QBN-$\mu_{i}$, QBN-$\theta_{i}$ form the QBN set for the quality bottleneck stage $M_{i}$. Among the QBN set, the largest quality bottleneck parameter is denoted as the primary QBN (P-QBN). Thus, the P-QBN of a multi-stage production system will be the one satisfying


(43) $$\max\left\{\mathrm{QBN}\text{-}\gamma_{i},\mathrm{QBN}\text{-}\eta_{i},\mathrm{QBN}\text{-}\mu_{i},\mathrm{QBN}\text{-}\theta_{i}\right\}$$

Improvements of parameter P-QBN in the quality bottleneck stage will bring in largest improvements for product quality.

## 6. Case Study

A case study in the production line of the valve shell was implemented to verify effectiveness. For data confidentiality, system parameters introduced to this case have modifications, while the “nature” for system parameters and structural properties hold. (1) Experimental setup: definition of system quality states, data collection, and calculation of quality transition probability. (2) Modeling: evaluation of quality performance and validation with measured data. (3) Structural property analysis and quality improvement: monotonicity and sensitivity analysis of system parameters, identification of quality bottleneck, and guidance for quality improvement.

### 6.1. Experimental Setup

**Figure 11:**
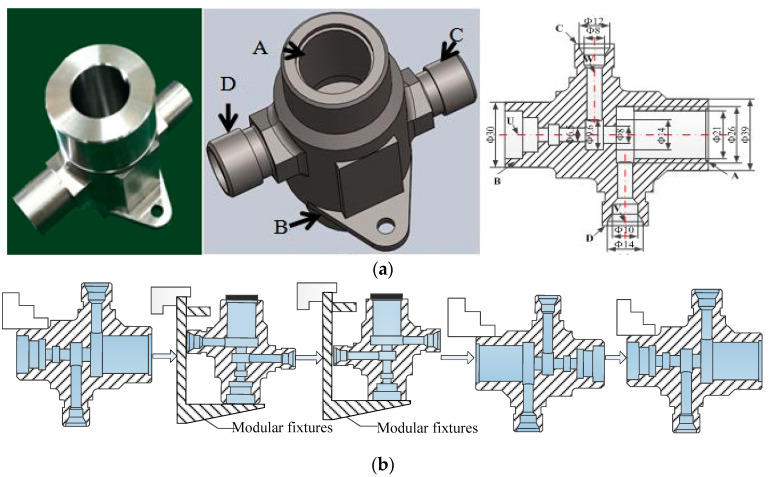
(**a**) Profiles of valve shell. (**b**) Production process.

The three-dimensional profiles of the valve shell are presented in Figure 11. OP 10 is the operation of processing Excircle Φ39 and Hole Φ21; OP20 processing Hole Φ10 and Hole Φ14; OP30 processing Hole Φ8 and Hole Φ12; OP40 processing Excircle Φ30, Hole Φ6.5, and Slot Φ9.6; OP50 processing Hole Φ8, Slot Φ14, and Slot Φ26, respectively.

These five stages are correlated and interconnected. For instance, the quality of the hole processed at OP10 may be corrected or degrade at downstream OP50. Moreover, flatness variations for the shell end face processed by upstream OP10 may influence the accuracy of clamping in stage OP20 and the downstream stage OP30. The product quality processed after stage $M_{i}\left(i\geq 2\right)$ depends on not only states in $M_{i}$, but also incoming product quality from upstream $M_{i-1}$. The incoming product before processing for every stage is of defective or good quality. Both quality corrections and quality degradations exist at the production system.

By implementing processing data analysis, state transition probability data are estimated on the factory floor. Record the quality of a certain product k before processing in stage $M_{i}$, and mark it as defective or good. After it is processed in stage $M_{i}$, again, record the product quality, and mark it as defective or good. For product k manufactured in stage $M_{i-1}$, it may be a defective or good incoming product for downstream $M_{i}$. The last product $\left(k-1\right)$ after manufactured in $M_{i}$ may also be defective or good. In case of a good or defective incoming product, after product k is manufactured in $M_{i}$, there are four possible situations of stage $M_{i}$.

(1) Last product $\left(k-1\right)$ after manufactured in $M_{i}$ is good, and product k is good.
(2) Last product $\left(k-1\right)$ after manufactured in $M_{i}$ is good, and product k is defective.
(3) Last product $\left(k-1\right)$ after manufactured in $M_{i}$ is defective, and product k is good.
(4) Last product $\left(k-1\right)$ after manufactured in $M_{i}$ is defective, and product k is defective.

In case of a good incoming product, the percentage for situation (2) defines transition probability $\alpha_{1}$ for $M_{1}$ or $\gamma_{i}$ for $M_{i}\left(i\geq 2\right)$. The percentage for situation (3) defines transition probability $\beta_{1}$ for $M_{1}$ or $\mu_{i}$ for $M_{i}\left(i\geq 2\right)$. In case of a defective incoming product, percentages for situations (2) or (3) will be defined as $\eta_{i}$ or $\theta_{i}$ respectively. Calculate the percentage of change from one state to another, and quality repair and failure probabilities are obtained.

### 6.2. Modeling of Quality Performance

The transition probability data are illustrated, of quality repair probability and quality failure probability, statistically. $\alpha_{1}=0.05$, $\beta_{1}=0.9$, $\gamma_{i}=\left[0.05,\;0.1,\;0.05,\;0.05\right]$, $\eta_{i}=\left[0.5,\;0.5,\;0.4,\;0.5\right]$, $\mu_{i}=\left[0.8,\;0.8,\;0.9,\;0.9\right]$, $\theta_{i}=\left[0.4,\;0.3,\;0.2,\;0.4\right]$.

**Figure 12:**
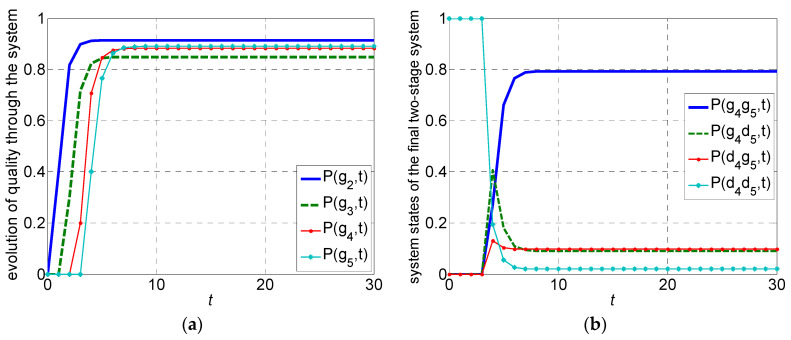
The evolutions for system quality performance during transients in the case: (**a**) product quality through the system, (**b**) system quality states at the last stage.

Using the probability data and the developed transient quality analysis method, the evolutions for system quality performance, quality loss, settling time, and steady-state quality are calculated. The steady-state quality at every two-stage aggregated system through the production line is, respectively, 91.37%, 84.86%, 88.24%, 89.06%. The final product quality of the measured data is 89.42%. The modeling error is 0.36%. Settling time is eight time slot. The dynamics for product quality $P\left(g_{i},t\right)$ at every two-stage system during the transients is presented in Figure 12a. The dynamics for system quality states at the last two-stage system $M_{4}^{\prime }-M_{5}$ is presented in Figure 12b. The calculation can clearly track system dynamics during transients. The results are consistent with measured data and validate the effectiveness of the developed approach.

**Figure 13:**
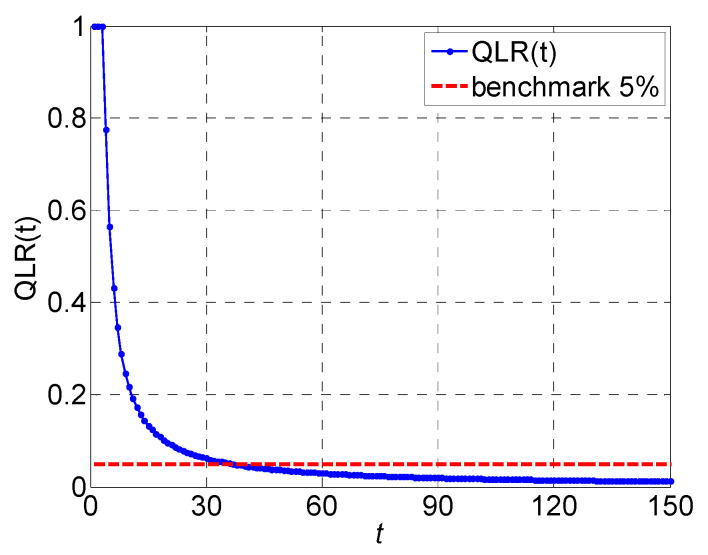
Quality loss rate curve over 150 time slots (the red dashed line is the benchmark of 5%).

$QLR\left(t\right)$ curve of the five-stage system is plotted over 150 time slots in Figure 13, where the red dashed line is the benchmark of 5%. $QLR\left(t\right)$ is quite significant during warming, which cannot be neglected. It diminishes gradually as time evolves. $QLR\left(t\right)$ will approximately diminish to zero when the production time horizon is sufficiently long. Moreover, the speed of convergence is much slower when the time is longer. Generally, in a practical production environment, a quality loss rate of 5% is considered as the upper limitation. Figure 13 also depicts that $QLR\left(t\right)$ decreases to below the 5% benchmark after 37 time slots, indicating that production system meets the criterion when the planned production time horizon is beyond 37 time slots. The effects of the quality loss rate should be taken into consideration when designing short-term production tasks in a practical production environment.

### 6.3. Structural Property Analysis and Quality Improvement

We first identify the quality bottleneck stage and then figure out the primary quality bottleneck parameter. The quality loss through each stage of the system is calculated as 0.8763, 1.0438, 1.5282, 1.7821, and 1.5539, respectively. The quality loss change after stage $M_{i}$ is calculated.

$$\begin{array}{c} \Delta L_{Q}(S_{1}(0))=L_{Q}(S_{1}(0))-L_{Q}(S_{2}(0))=-0.1675\\ \Delta L_{Q}(S_{2}(0))=L_{Q}(S_{2}(0))-L_{Q}(S_{1}(0))=0.1675\\ \Delta L_{Q}(S_{3}(0))=L_{Q}(S_{3}(0))-L_{Q}(S_{2}(0))=0.4844\\ \Delta L_{Q}(S_{4}(0))=L_{Q}(S_{4}(0))-L_{Q}(S_{3}(0))=0.2539\\ \Delta L_{Q}(S_{5}(0))=L_{Q}(S_{5}(0))-L_{Q}(S_{4}(0))=-0.2282 \end{array}$$

OP30 with the largest positive value is identified as the quality bottleneck stage since quality loss undergoes the largest increase in this stage. In the next step, we investigate which parameter will bring the maximum quality benefit to OP30 by changing only a parameter in the bottleneck stage at one time and keeping other parameters unchanged through the monotonic and sensitivity analyses.

**Figure 14:**
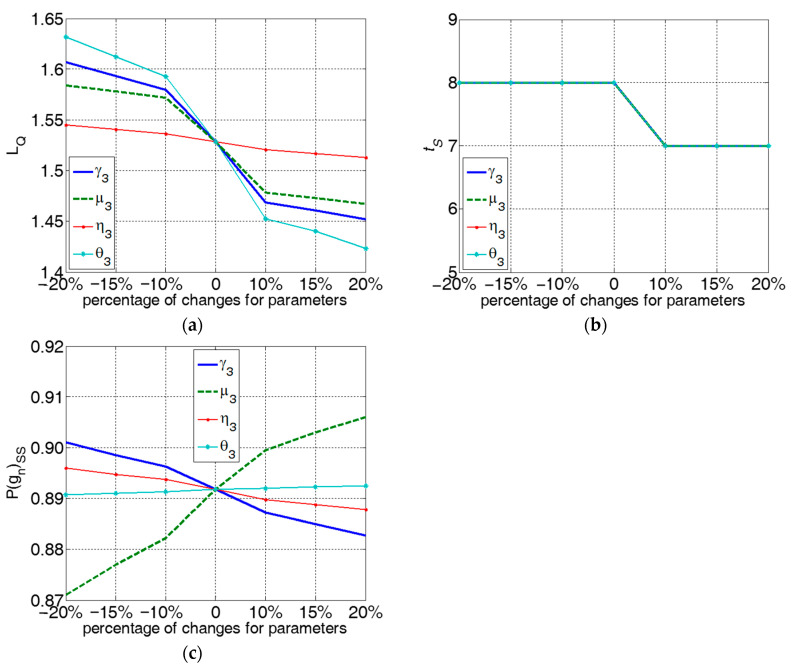
Change of (**a**) quality loss, (**b**) settling time, and (**c**) steady-state quality corresponding to the parameter change.

The parameters of OP30, i.e., $\gamma_{3},\eta_{3},\mu_{3},\theta_{3}$ decrease or increase with defined percentages. In particular, these parameters will be changed with ±10%, ±15%, ±20%. Quality loss, settling time, and steady-state quality corresponding to the parameter changes are calculated respectively and presented in Figure 14.

(1) From Figure 14a, the monotonic property for quality loss is in accordance with numerical results 3–4. Quality loss is decreased when system parameters of OP30 increase. According to the sensitivity analysis in the quality bottleneck stage, parameters QBN-$\gamma_{3}$, QBN-$\mu_{3}$, QBN-$\eta_{3}$, QBN-$\theta_{3}$ form the QBN set for OP30 with values {0.3900, 0.3285, 0.0510, 0.4947}. QBN-$\theta_{3}$ is denoted as the P-QBN. Quality loss in OP30 is most sensitive regarding quality repair probability in case of a defective incoming product $\theta_{3}$. Proper changes of $\theta_{3}$ will bring the largest reduction to quality loss $L_{Q}\left(S_{3}\left(0\right)\right)$ and prevent OP30 from being the quality bottleneck stage.
(2) From Figure 14b, monotonic property for settling time is consistent with numerical results 1–2. The settling time will be reduced when the system parameter increases. As shown in Figure 6 and Figure 7, since settling time is eight or seven time slots in the range sets of transition probability given above, the four curves regarding parameters overlap in this case study.
(3) From Figure 14c, monotonic property for the steady-state quality is consistent with numerical result 5. Steady-state quality will improve when $\mu_{3}$ or $\theta_{3}$ increases, and when $\gamma_{3}$ or $\eta_{3}$ decreases. In the sensitivity analysis, $\mu_{3}$ is the most sensitive parameter. Improving quality repair probability in case of a good incoming product $\mu_{3}$ achieves a better steady-state quality.
  The most sensitive parameter of steady-state quality is viewed as the quality bottleneck parameter in a steady-state phase. Correspondingly, the QBN set and P-QBN are viewed as quality bottleneck parameters in the transient phase. In some cases, the transient bottleneck parameter and steady-state bottleneck parameter are just the same parameter. However, in other cases, the two parameters may be different. As shown in the case study, they are $\theta_{3}$ and $\mu_{3}$ respectively.
(4) When transient and steady-state quality bottleneck parameters fall in the same parameter, there is a desire to improve this particular parameter to facilitate quality performance in both the transient and steady-state regime. When they fall in different parameters, we can attempt to seek a balance between transients and the steady-state. Firstly, if production time horizon is relatively long, or when designing long-term production systems, we should focus on the steady-state quality bottleneck parameter since transients can be neglected compared with the overall production; on the contrary, we may focus on the transient quality bottleneck parameter. Secondly, if the criterion of quality loss rate is high, focus on the transient bottleneck parameter to prioritize reduction in quality loss; contrarily, focus on the steady-state bottleneck parameter.

To summarize, transient analysis of quality in a five-stage production line provides insight in regard to improving product quality. Increase of $\theta_{3}$ of OP30 will shorten the transient duration and decrease quality loss during transients, while increasing $\mu_{3}$ is favorable for steady-state quality.

## 7. Conclusions

A novel analytical PSM approach was established based on the Markov model, to explore product quality propagation for transient analysis of serial multi-stage production systems with RQIF. The cascade property for quality propagation among the correlated sequential stages was investigated, taking into account both the status of the current stage and quality of the outputs from upstream stages. Closed-form formulae to evaluate transient quality performances of multi-stage systems were formulated. An iterative procedure utilizing the aggregation technique was presented to approximate the transient quality performance with computational efficiency and high accuracy. Moreover, system theoretic properties of quality measures were analyzed and the quality bottleneck identification method was investigated. In the case study, the modeling error was 0.36% and the calculation could clearly track system dynamics, quality bottleneck was identified to decrease quality loss and facilitate continuous improvement. The experimental results illustrate the applicability of the proposed PSM approach. This paper paves the way for modeling, analyses, and improvements for the system quality performances of serial multi-stage production systems in transient phases.

The contributions of the proposed approach are summarized from these aspects. Firstly, compared to conventional Markov models and quality flow models, this approach overcomes the assumption that stages are independent, and the restriction that each stage has an inspection and repair station. Both quality corrections and quality degradations were addressed, and they are more practical and accurate in a real production environment. This enables PSM to be more promising with wide applicability for quality modeling of production systems. Secondly, existing analytical PSM research can only deal with a steady-state performance, while the proposed approach can characterize both steady-state and transient quality behavior. Based on the Markov model and probability theory, a transient quality analysis was conducted to reveal the correlation between the components and system quality performance. Thirdly, system theoretic properties of critical quality measures during transients were thoroughly analyzed. The quality bottleneck identification method was derived in terms of the quality bottleneck stage and parameter. A numerical analysis provides directions pertaining to resource optimization and continuous quality improvement for plant managers.

Future research can focus on the following issues: (1) the extension of the proposed method to assembly systems and other production systems with more complicated structures. (2) The investigation of a real-time quality performance feedback control to meet the demands on energy efficiency in current smart production trends. (3) Transient analysis of multi-type product production systems.

## Data Availability

The data in this paper is available upon requests.

## Notations

*M_i_*	*i*th stage of multi-stage production systems
*M_i_*′	the merged stage from the first *i* stages of multi-stage production systems
*g_i_*	*M_i_* or *M_i_*′ produces a good product
*d_i_*	*M_i_* or *M_i_*′ produces a defective product
*α*_1_	probability of *M*_1_ transiting from *g*_1_ to *d*_1_
*β*_1_	probability of *M*_1_ transiting from *d*_1_ to *g*_1_
*α_i_*′	probability of *M_i_*′ transiting from *g_i_* to *d_i_*
*β_i_*′	probability of *M_i_*′ transiting from *d_i_* to *g_i_*
*γ_i_*	in case of good coming product, probability of *M_i_* transiting from state *g_i_* to *d_i_*
*μ_i_*	in case of good coming product, probability of *M_i_* transiting from state *d_i_* to *g_i_*
*η_i_*	in case of defective coming product, probability of *M_i_* transiting from state *g_i_* to *d_i_*
*θ_i_*	in case of defective coming product, probability of *M_i_* transiting from state *d_i_* to *g_i_*
*g_i_g_i+_*_1_	*M_i_* or *M_i_*′ produces good product, *M_i+_*_1_ produces good product
*g_i_d_i+_*_1_	*M_i_* or *M_i_*′ produces good product, *M_i+_*_1_ produces defective product
*d_i_g_i+_*_1_	*M_i_* or *M_i_*′ produces defective product, *M_i+_*_1_ produces good product
*d_i_d_i+_*_1_	*M_i_* or *M_i_*′ produces defective product, *M_i+_*_1_ produces defective product
*S_i_(t)*	state probability matrix with *i* stage at time slot *t*
*C_i_*	state transition probability matrix with *i* stage
*P(g_i_t)*	probability of producing good product with *i* stage at time slot *t*
*P(g_i_)_ss_*	probability of producing good product with *i* stage in steady-state
*t_s_*	settling time for system quality to approach steady-state
*L_Q_*	quality loss during transients
*QLR(t)*	quality loss rate over *t* time slots
*QBN–*	quality bottleneck parameters
